# NDRG2 suppresses the proliferation of clear cell renal cell carcinoma cell A-498

**DOI:** 10.1186/1756-9966-29-103

**Published:** 2010-07-30

**Authors:** Jian-Jun Ma, Cheng-Gong Liao, Xue Jiang, Hua-Dong Zhao, Li-Bo Yao, Ting-Yi Bao

**Affiliations:** 1 Department of Urology, Tangdu Hospital, the Fourth Military Medical University, Xi'an, China; 2 Cell Engineering Research Centre, the Fourth Military Medical University, Xi'an, China; 3 Department of Operation Room, Tangdu Hospital, the Fourth Military Medical University, Xi'an, China; 4 Department of General Surgery, Tangdu Hospital, the Fourth Military Medical University, Xi'an, China; 5 Department of Biochemistry and Molecular Biology, the Fourth Military Medical University, Xi'an, China

## Abstract

**Background:**

Recently, the anti-tumor activity of N-myc downstream-regulated gene 2 (NDRG2) was shown decreased expression in clear cell renal cell carcinoma (CCRCC), but the role of the down-expression of NDRG2 has not been described.

**Methods:**

The NDRG2 recombinant adenovirus plasmid was constructed. The proliferation rate and NDRG2 expression of cell infected with recombinant plasmid were mesured by MTT, Flow cytometry analysis and western blot.

**Results:**

The CCRCC cell A-498 re-expressed NDRG2 when infected by NDRG2 recombinant adenovirus and significantly decreased the proliferation rate. Fluorescence activated cell sorter analysis showed that 25.00% of cells expressed NDRG2 were in S-phase compared to 40.67% of control cells, whereas 62.08% of cells expressed NDRG2 were in G1-phase compared to 54.39% of control cells (*P *< 0.05). In addition, there were much more apoptotic cells in NDRG2-expressing cells than in the controls (*P *< 0.05). Moreover, upregulation of NDRG2 protein was associated with a reduction in cyclin D1, cyclin E, whereas cyclinD2, cyclinD3 and cdk2 were not affected examined by western blot. Furthermore, we found that p53 could upregulate NDRG2 expression in A-498 cell.

**Conclusions:**

We found that NDRG2 can inhibit the proliferation of the renal carcinoma cells and induce arrest at G1 phase. p53 can up-regulate the expression of NDRG2. Our results showed that NDRG2 may function as a tumor suppressor in CCRCC.

## Background

Renal cell carcinoma (RCC) accounts for 3% of all malignant tumors and 90% of neoplasms arising from the kidney. The incidence rates vary more than 10-fold around the world; rates are higher in Western countries than in Asia. In the United States, renal cancer is the 7th leading malignant condition among men and the 12th among women [1]. Clear cell renal cell carcinoma (CCRCC) originates from proximal tubule cells and is the most common pathological type of renal cell carcinoma. Multiple genetic changes have been found in CCRCC, but little is known about major tumor suppressor genes involved in the tumorigenesis of the disease.

N-myc downstream regulated gene 2 (NDRG2) belongs to the NDRG family, which is comprised of 4 members, NDRG1-4, and is expressed in the tissues of the brain, heart, skeletal muscle, and kidney [2]. NDRG2 was identified through sequence homology and is implicated in cell growth, differentiation and neurodegeneration [3-6]. It has been proposed that NDRG2 is a candidate tumor suppressor gene since it induces apoptosis in certain cancer cells and mRNA was down-regulated or absent in several human cancers and cancer cell-lines [3,7,8]. In addition, higher expression of NDRG2 mRNA correlated with clinically less aggressive tumors in meningiomas [8] and NDRG2 expression in high-grade gliomas was positively correlated with survival [9].

Until now, a mechanism for the inactivation of NDRG2 in cancer cells has not been described. In previous studies, we found that the expression level of NDRG2 mRNA and protein were down-regulated in renal tissue and CCRCC [10], indicating that NDRG2 might play an important role in the carcinogenesis and development of CCRCC. In the present work, we found that forced expression of NDRG2 can inhibit the proliferation of the renal carcinoma cells and induce arrest at G1 phase. p53 can up-regulate the expression of NDRG2. Our results showed that NDRG2 may function as a tumor suppressor in CCRCC.

## Methods

### Construction of recombinant adenovirus

The 1.2 kb NDRG2 gene was released from pET44a-NDRG2 plasmid (provided by Dr. Wei Zhang) by Sal I---Hind III restriction enzyme digestion, and inserted into the same site of plasmid pAdTrack-CMV, resulting in plasmid pAdTrack-NDRG2.

pAdTrack-NDRG2 was linearized with PmeI and transfected into Escherichia coli BJ5183 cells together with pAdEasy-1 (Stratagene Holding Corporation, La Jolla, CA, USA) by electroporation, and the recombinants were selected with kanamycin. The clonies were picked, grown, and then plasmids were extracted, screened and analyzed by agarose gel electrophoresis, and one named AdEasy-GFP-NDRG2 selected. The construction of recombinant adenovirus AdEasy-GFP-NDRG2 was performed as described by Tran et al [11]. Infectious viruses were purified by plaques. All recombinant adenoviruses were amplified on human embryonic kidney cell line 293 and purified by double cesium chloride density gradient ultracentrifugation. Titers of the adenoviral stocks were determined by plaque assay on 293 cells. Photograph of viral plaque formation to count viral titer (plaque assay). HEK-293 cells, which grew confluently on the bottom of the 24-well plastic plate (1.5 cm diameter each), were infected with serially diluted solutions containing adenoviral virus, and then cultured over night to make viral plaque. The number of plaques indicates the number of the infectious virus (= viral titer, as plaque forming unit). AdEasy-GFP-p53 was provided by Dr. Lintao Jia.

### Cell Culture

The human renal clear-cell carcinoma lines A-498 and the human embryonic kidney cell lines HEK-293 were obtained from the American Type Culture Collection (ATCC) and maintained as recommended. A-498 was cultured in Minimum Essential Medium (MEM) with 2 mM L-glutamine and Earle's BSS adjusted to contain 1.5 g/l sodium bicarbonate, 0.1 mM non-essential amino acids, and 1.0 mM sodium pyruvate. HEK-293 was cultured with Dulbecco's Modified Eagles' Medium (DMEM). All the culture fluid was supplemented with 10% fetal calf serum (FCS) and all cells were cultured with 5% CO_2 _at 37°C in a humidified chamber.

### Western blot analysis

Cells were washed with ice-cold PBS and lysed in a RIPA buffer [50 mM Tris (pH7.5), 150 mM NaCl, 1% NP-40, 0.5% sodium deoxycholate, 0.1% SDS] containing PMSF (1 mM) and protease inhibitors (2 μg/ml; Protease Inhibitor Cocktail Set III, Calbiochem) on ice for 30 minutes. The lysates were clarified by centrifugation at 13,000 × g for 30 minutes at 4°C. The total protein concentration was estimated using Protein Assay Kit (Bio-Rad, Richmond, CA). 30-80 μg protein samples were loaded on a 12% SDS-PAGE and subsequently transferred to polyvinylidene difluoride membranes. After being blocked with TBST [20 mM Tris (pH7.5), 150 mM NaCl, 0.01% Tween-20] containing 5% non-fat dry milk for 1 hour at room temperature, membranes were probed with an appropriate antibody overnight at 4°C followed by a horseradish peroxidase (HRP)-linked goat anti-mouse or anti-rabbit antibodies at room temperature for 1 hour. The membranes were analyzed using super ECL detection reagent (Applygen, Beijing, China).

The following antibodies were used: NDRG2 (dilution 1:500; prepared by Department of Immunology, FMMU) [12], cyclin D1, cyclin E, cyclin D2, cyclin D3 and cyclin-dependent kinase (cdk) 2 (dilution 1:200, Santa Cruz Biotechnology, Santa Cruz, CA), p53 (dilution 1:500; BD Biosciences, San Jose, CA), anti-β-actin (dilution 1:5000, Sigma Chemical Company, St. Louis, MO).

### Cell survival assays

Briefly, cells were seeded at an initial density of 5 × 10^4 ^cells/ml in a 96-well plate for 24 h. After transfection, MTT (3-(4,5-Dimethylthiazol-2-yl)-2,5-diphenyltetrazolium bromide) was added into each well at a final concentration of 0.5 mg/ml. The insoluble formazan was collected, dissolved in dimethylsulfoxide and measured with an ELISA reader (Bio-Rad, USA) at a wavelength of 570 nm.

### RNA isolation and reverse transcription-polymerase chain reaction (RT-PCR)

RNA isolation was performed using TRIzol reagent (Invitrogen, Carlsbad, CA) according to the manufacture's protocol. SuperScript Preamplification System (Gibco BRL, Gaithersburg, MD) was used for cDNA synthesis. Two microgramme of cDNA was used as a template for PCR reaction. The following primers were used: GAPDH: forward 5'-GTC AGT GGT GGA CCT GAC CT-3' and reverse 5'-AGG GGT CTA CAT GGC AAC TG-3'; p53: forward 5'-TAC TCC CCT GCC CTC AAC AAG A -3' and reverse 5'-CTT AGC ACC TGA AGG GTG AAA TAT TC-3', and NDRG2: forward 5'- ATG GCG GAG CTG CAG GAG GTG-3' and reverse 5'-AAC AAG GGC CAT TCA ACA GGA GAC-3'. The cycling conditions were as follows: initial denaturantion (5 minutes at 94°C), followed by the appropriate number of 26 cycles of denaturation (94°C, 30 seconds), annealing (GAPDH, 30 seconds at 60°C; p53, 30 seconds at 65°C; NDRG2, 30 seconds at 68°C) and elongation (30 seconds at 72°C), and a final extension (10 minutes at 72°C). The samples were visualized by electrophoresis in 1.2% agarose gel and ethidium bromide.

### Cell Cycle and apoptosis Analysis

Flow cytometry analysis was performed as described. Cells were seeded overnight on 60-mm-diameter plates in a complete medium, placed in a serum-free medium for 48 hours to synchronize the cells, and then kept again in the complete medium. At 24 hours, cells were recovered. After washing with ice-cold PBS, cells were suspended in about 0.5 ml of 70% alcohol and kept at 4°C for 30 minutes. The suspension was filtered through a 50-mm nylon mesh, and the DNA content of stained nuclei was analyzed by a flow cytometer (EPICS XL; Coulter, Miami FL). Cell cycle was analyzed using Multicycle-DNA Cell Cycle Analyzed Software (FACScan, Becton Dickinson, San Jose, CA). The proliferous index (PI) was calculated as: PI = (S + G2)/(S + G2 + G1). Apoptosis index was measured using Annexin V-FITC apoptosis detection kit (Sigma) and subsequently analyzed by flow cytometry. Each experiment was performed in triplicate [13,14].

### Statistical Analysis

All statistical analyses were performed using the SPSS 16.0 statistical software package (SPSS, Chicago, IL). The differences in apoptosis index between groups were compared using one-way analysis of variance, and data were expressed as mean ± SEM. Statistical difference was accepted at *P *< 0.05.

## Results

### Validation of recombinant adenovirus

**Figure 1:**
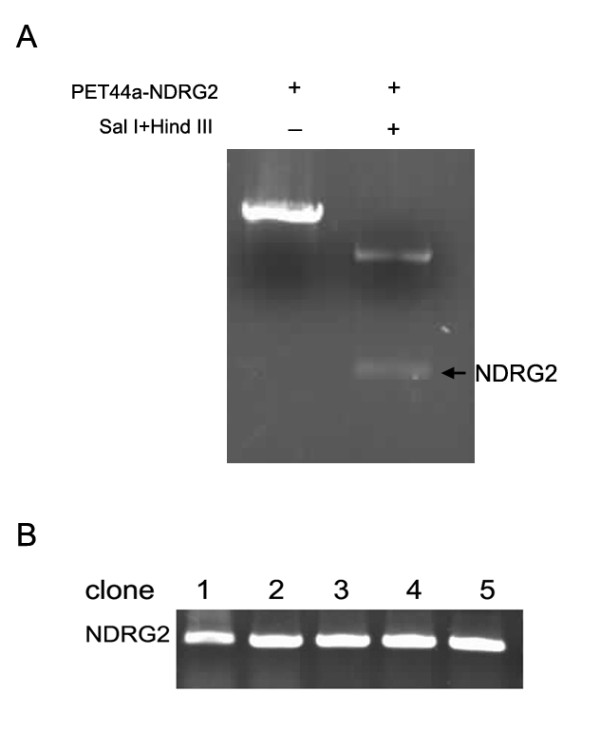
**Validation of recombinant adenovirus**. (A) The pET44a-NDRG2 plasmid with and without digestion by by Sal I---Hind III restriction enzyme were shown. (B) The PCR product of target segment in AdEasy-GFP-NDRG2.

The pET44a-NDRG2 plasmid and 1.2 kb NDRG2 gene released from plasmid by Sal I---Hind III restriction enzyme digestion were shown in Fig. 1A. The target segment in AdEasy-GFP-NDRG2 was detected by PCR. Results of electrophoresis on PCR amplification of the target segment in AdEasy-GFP-NDRG2 are shown in Fig. 1B. Five clones were picked. Titers of the adenoviral stocks were 3.1 × 10^8 ^cfu/ml.

### NDRG2 Inhibits CCRCC cell Proliferation

**Figure 2:**
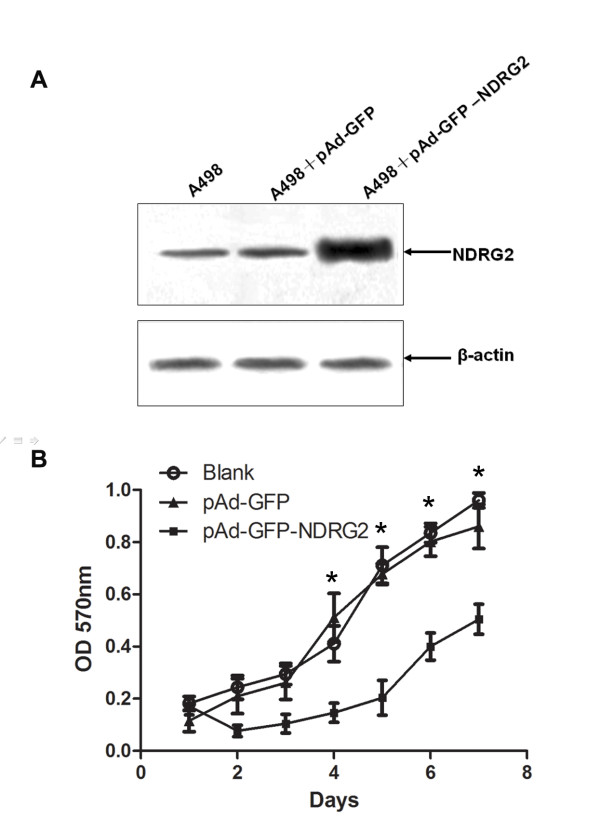
**NDRG2 inhibits the proliferation of CCRCC cells**. (A) Tthe protein expression was detected by Western blotting. (B) The proliferation of A-498 cells was detected by MTT.* *P *< 0.05.

To elucidate the functional role of NDRG2 in renal tumorigenesis, we examined the effect of exogenous expression of NDRG2 on the malignant phenotype of CCRCC cells, A-498. Western blotting revealed that A-498 expressed NDRG2 when infected by recombinant adenovirus pAd-GFP-NDRG2 (Fig. 2A).

We then tested the effect of NDRG2 on the Proliferation of A-498 cells. Growth curves were compared in a medium containing 10% fetal calf serum, the curves for cells expressed NDRG2 was significantly lower than those for control cells(*P *< 0.05; Fig. 2B). This suggested that NDRG2 had the potential to inhibit the proliferation of CCRCC cells.

### NDRG2 Induces the Cell Cycle Arrest and apoptosis of CCRCC Cells

**Figure 3:**
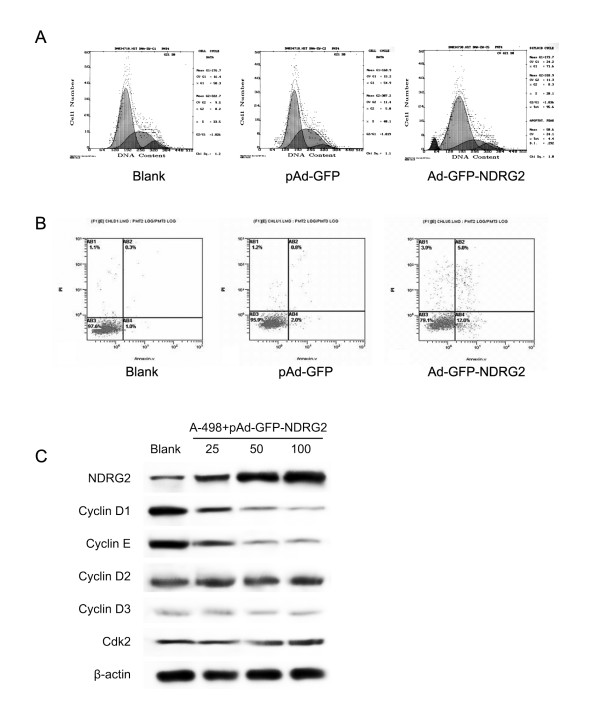
**NDRG2 Induces the Cell Cycle Arrest and apoptosis of CCRCC Cells**. (A) and (B) The effects of NDRG2 expression on the cell cycle and apoptosis were detected by FASC. (C) The cell cycle protein were examined by western blot analysis.

To further investigate the mechanism by which NDRG2 inhibits CCRCC cell growth, we studied the effects of NDRG2 expression on the cell cycle by fluorescence activated cell sorter analysis (FASC). The results of the cell cycle showed that 25.00% of cells expressed NDRG2 were in S-phase compared to 40.67% of control cells, whereas 62.08% of cells expressed NDRG2 were in G1-phase compared to 54.39% of control cells (*P *< 0.05, Fig. 3A). In addition, FASC also revealed that there were much more apoptotic cells in NDRG2 -expressing cells than in the controls (*P *< 0.01, Fig. 3B). We then investigated the mechanism by which NDRG2 induced cell cycle arrest in CCRCC cells. Cell cycle effectors were examined by western blot analysis (Fig. 3C). Our results indicated that upregulation of NDRG2 protein was associated with a reduction in cyclin D1, cyclin E proteins, whereas cyclinD2, cyclinD3 and cdk2 were not affected.

### p53 up-regulates NDRG2 expression in CCRCC cells

**Figure 4:**
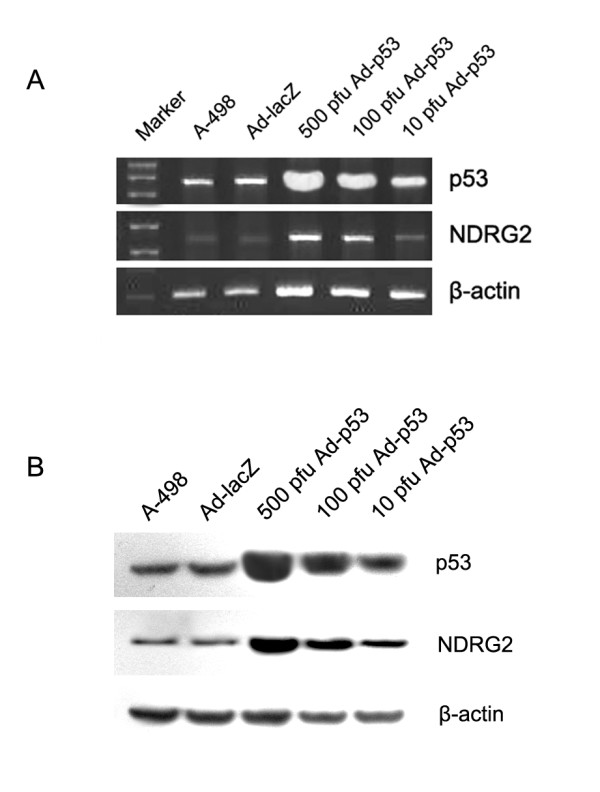
**p53 up-regulates NDRG2 expression in CCRCC cells**. (A) and (B) RT-PCR and Western blot analysis were used to detect the p53 and NDRG2 mRNA and protein expression levels. Ad-lacZ was used as negative control.

Bioinformatics analysis suggested that there was a p53 binding site in upstream of NDRG2 promoter. To investigate whether NDRG2 expression was regulated by p53, we first infected A-498 cells with recombinant adenovirus Ad-p53. Both RT-PCR and Western blot revealed that A-498 cells expressed p53 at higher level when infected transfected with Ad-p53 (Fig. 4). We then examined NDRG2 expression in these cells. NDRG2 mRNA was very low in A-498 or cells transfected with Ad-lacZ but was highly upregulated in cells expressed Ad-p53, and this upregulation was dose dependent. Western blot confirmed that NDRG2 protein was upregulated by p53 (Fig. 4).

## Discussion

The treatment of renal cancer is challenging due to its strong resistance to conventional cancer therapy. The development and progression of RCC is thought to mainly arise from changes in some key genes that are related to cell proliferation, apoptosis and genomic stability. Therefore, it is important to identify more genes specifically related to renal cell carcinoma, which may expand our understanding of this disease and assist in the development of new targets for the therapy and diagnostic indicators. In our previous studies, NDRG2 positive expression found in CCRCC specimens was 30.3% (40/132), which was significantly lower than the 91.67% (121/132) in their adjacent tissues. These data indicated that decreased of NDRG2 expression is a frequent event in human renal cell carcinoma.

To determine whether the ectopic expression of NDRG2 could modulate the proliferation of renal cancer cells, duplication-defective adenovirus was used as the vehicle. The results of verification showed that the NDRG2 effectively incorporated into the plasmid of the recombinant adenovirus. This recombinant adenovirus had a high transfection on A-498 renal cancer cells and successfully expressed NDRG2 at a high level. We found that NDRG2 significantly inhibited renal cancer cell proliferation. Then we demonstrated that NDRG2 tumor-suppressor activity is mediated by the inhibition of cell cycle progression with increased accumulation of cancer cells in G1-phase and a corresponding reduction of cells in the S-phase of the cell cycle in the A-498 renal cancer cells. Very recently, Kim et al. reported that NDRG2 suppressed cell proliferation through down-regulation of AP-1 activity in human colon carcinoma cells[15]. They found that NDRG2 modulated intracellular signals to control cell cycle through the regulation of cyclin D1 expression via phosphorylation pathway, which might helped to explain alterations of cell cycle effectors in our research.

Also clearly in our studies, NDRG2 induced renal cancer cell apoptosis. NDRG2 was lately reported to be involved in hypoxia-induced apoptosis or fas-mediated cell death in different cancer cell types [16,17]. Investigations carried out by Liu et al. indicated that NDRG2 was a new target gene that is regulated by p53 and NDRG2 mRNA and protein levels can be upregulated in a p53-dependent manner [13,18], which were also observed in our work. They further reported that silencing of NDRG2 attenuates p53-mediated apoptosis. These data strongly suggested that NDRG2 was an important factor in regulating tumor cell apoptosis.

## Conclusions

Our results show that enforced NDRG2 expression significantly inhibited RCC cell growth, and induced apoptosis in human renal carcinoma cells. We also observed that NDRG2 expression could be upregulated by p53 in dose dependent manner. Further research may help design an effective therapeutic modality to control renal cancer.

## Abbreviations

NDRG2: N-myc downstream-regulated gene 2; CCRCC: clear cell renal cell carcinoma; RT-PCR: Reverse transcription-polymerase chain reaction.

## Competing interests

The authors declare that they have no competing interests.

## Authors' contributions

TYB and LBY contributed to the conception and design of the study; JJM performed research; XJ and HDZ contributed to collection and assembly of data; JJM and CGL contributed to data analysis and manuscript writing. All authors have read and approved the final manuscript.
